# Contemporary survival of patients with pulmonary arterial hypertension and congenital systemic to pulmonary shunts

**DOI:** 10.1371/journal.pone.0195092

**Published:** 2018-04-17

**Authors:** Chodchanok Vijarnsorn, Kritvikrom Durongpisitkul, Paweena Chungsomprasong, Densiri Bositthipichet, Salisa Ketsara, Yuttapon Titaram, Prakul Chanthong, Supaluck Kanjanauthai, Jarupim Soongswang

**Affiliations:** Faculty of Medicine Siriraj Hospital, Mahidol University, Bangkok, Thailand; Kurume University School of Medicine, JAPAN

## Abstract

**Objective:**

To compare survival of patients with newly diagnosed pulmonary arterial hypertension associated with congenital heart disease (PAH-CHD) according to various clinical classifications with classifications of anatomical-pathophysiological systemic to pulmonary shunts in a single-center cohort.

**Methods:**

All prevalent cases of PAH-CHD with hemodynamic confirmation by cardiac catheterization in 1995–2015 were retrospectively reviewed. Patients who were younger than three months of age, or with single ventricle following surgery were excluded. Baseline characteristics and clinical outcomes were retrieved from the database. The survival analysis was performed at the end of 2016. Prognostic factors were identified using multivariate analysis.

**Results:**

A total of 366 consecutive patients (24.5 ± 17.6 years of age, 40% male) with PAH-CHD were analyzed. Most had simple shunts (85 pre-tricuspid, 105 post-tricuspid, 102 combined shunts). Patients with pre-tricuspid shunts were significantly older at diagnosis in comparison to post-tricuspid, combined, and complex shunts. Clinical classifications identified patients as having Eisenmenger syndrome (ES, 26.8%), prevalent left to right shunt (66.7%), PAH with small defect (3%), or PAH following defect correction (3.5%). At follow-up (median = 5.9 years; 0.1–20.7 years), no statistically significant differences in survival rate were seen among the anatomical-pathophysiological shunts (*p* = 0.1). Conversely, the clinical classifications revealed that patients with PAH-small defect had inferior survival compared to patients with ES, PAH post-corrective surgery, or PAH with prevalent left to right shunt (*p* = 0.01). Significant mortality risks were functional class III, age < 10 years, PAH-small defect, elevated right atrial pressure > 15 mmHg, and baseline PVR > 8 WU•m.^2^

**Conclusion:**

Patients with PAH-CHD had a modest long-term survival. Different anatomical-pathophysiological shunts affect the natural presentation, while clinical classifications indicate treatment strategies and survival. Contemporary therapy improves survival in deliberately selected patients.

## Introduction

Pulmonary hypertension (PH) describes a number of different diseases involving vasculature pressure changes that affect hemodynamic and therapeutic features. Elevated mean pulmonary arterial pressure (mPAP) above 25 mmHg resulting in an increase in pulmonary vascular resistance (PVR) is the hallmark of the disease [1]. Pulmonary arterial hypertension (PAH) is a large subset of PH diseases that include idiopathic, heritable, congenital heart disease (CHD), connective tissue disease, human immunodeficiency virus, portal hypertension, and drugs and toxins, which are characterized by the presence of precapillary PH. Recently, these have been defined as having mPAP ≥ 25 mmHg at rest, evaluated by cardiac catheterization in the presence of normal pulmonary artery wedge pressure (PAWP) ≤ 15 mmHg, and a PVR > 3 Wood unit (WU) [2–4]. Congenital malformations of the heart, such as systemic to pulmonary shunt lesion are the most common diseases associated with PAH in response to a chronic volume overload of the pulmonary circulation in the early stages of life. If unrepaired, large defects and irreversible pathological changes to the pulmonary vessels may occur and high pulmonary vascular resistance may develop later with consequent reversal shunt and cyanosis, the so-called Eisenmenger syndrome (ES). Other cardiac malformations may lead to PH, such as left heart inflow and outflow obstruction, which is currently classified in PH as being due to left heart disease, and not as a PAH-CHD. PAH in the context of single ventricle physiology after staged palliation such as Fontan palliation is also recognized. This complex circulation may not fulfill the standard criteria for PAH but elevated PVR may lead to “failing Fontan circulation”. The prevalence of PAH associated with congenital heart disease (PAH-CHD) has risen in recent decades to 5–28% (ranging between 1.6 and 12.5 cases per million adults). Of these cases, 25–50% were classified as ES [5, 6]. This prevalence reflects the growing proportion of children with CHD who are reaching adulthood, indicating the accomplishments of advanced management of both PH and CHD.

Currently, clinical classifications and the anatomical-pathophysiological classifications provide detailed descriptions of individual patients with PAH-CHD. Clinical classifications divide PAH-CHD patients into four groups: ES, PAH associated with prevalent systemic to pulmonary shunts, PAH with small defect, and PAH after defect correction [2–4]. The anatomical-pathophysiological classifications that describe PAH-CHD are based on type, dimension, shunting direction, associated cardiac/extracardiac defects, and the repair status. The systemic to pulmonary shunts are categorized into four types: simple pre-tricuspid shunts, simple post-tricuspid shunts, combined shunts, and complex CHD [4, 7, 8]. Recently released guidelines also include pediatric PAH as a specific subset of PAH and adopt both classifications into clinical practice for treating PAH-CHD in children [4]. Prognosis and survival outcomes have been shown to be associated with the clinical classifications in some large adult cohort studies [9, 10] and the anatomical variance appears to be relevant to the survival of ES patients [10–12]. Ramjug and colleagues applied both types of classification to 240 PAH-CHD patients and found that the anatomical-pathophysiological classifications identified different survival rates [13]. In the modern era, PAH-specific therapy has been proposed and shown to alleviate symptoms in many forms of PAH, even in extreme form such as ES [12, 14]. In North America, Europe, Australia, and China, national PAH registries have been well established and the long-term data for case incidence of several forms of PAH has been reported [9, 10, 15–19]. Unfortunately, the cohort data from developing countries, where patients may have undiagnosed or un-operated CHD, is scarce [14, 20].

Faculty of Medicine Siriraj Hospital, one of the main referral hospitals in Thailand for congenital cardiac surgery and intervention, initiated the institute’s PH database to be a preliminary part of the national registry in 2012. All prevalent cases that were newly diagnosed and confirmed by cardiac catheterization in the center since 1995 were retrospectively reviewed and entered into a prospective cohort. The aims of the present study are to identify survival rates of newly diagnosed patients with PAH-CHD with various clinical classifications and classifications of anatomical-pathophysiological systemic to pulmonary shunts in a large referral center in Thailand.

## Materials and methods

**Fig 1:**
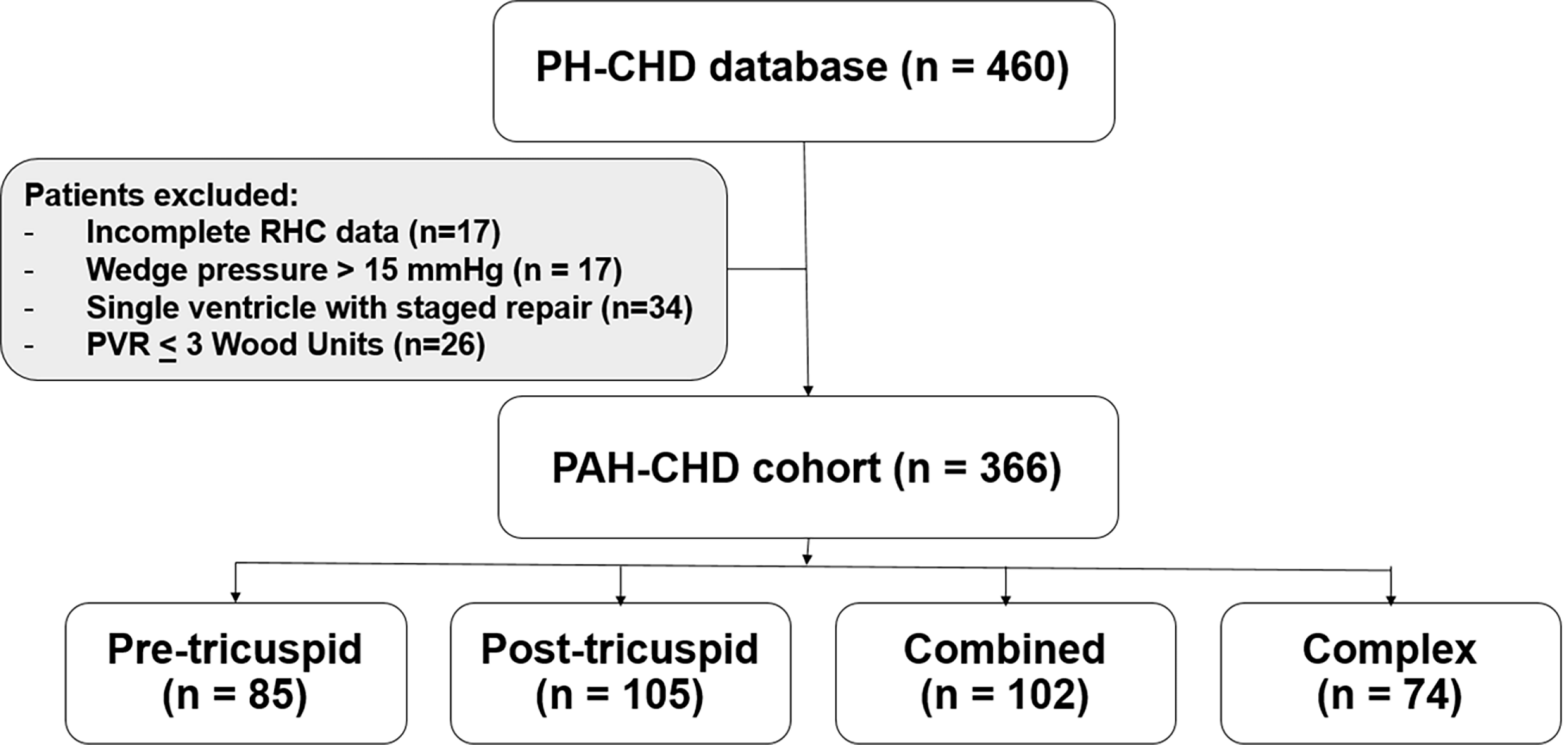
Flow of patient cases in the study. (PH, pulmonary hypertension; CHD, congenital heart disease; PAH, pulmonary arterial hypertension; RHC, right heart catheterization).

Following the approval of the Institutional Ethic Committee, Faculty of Medicine Siriraj Hospital, Mahidol University, the records of 460 patients with PH-CHD who had undergone cardiac catheterization to assess their hemodynamic data or transcatheter closure of the defects in Siriraj Hospital from January 1, 1995 to December 31, 2015 were retrospectively retrieved from the database. All patients with evidence of PAH on cardiac catheterization, including mPAP ≥ 25 mmHg, PAWP ≤ 15 mmHg, and calculated PVR > 3 WU based on the current definition of PAH [2–4] were included. Patients who were younger than three months of age or who were single ventricle following surgery were excluded from the study. A total of 366 patients (174 age < 18 years; 192 age ≥ 18 years) were identified from the database (Fig 1). Demographic information including date of birth, gender, presenting symptom, functional class, hematocrit (%), clinical classification (ES, PAH associated with prevalent systemic to pulmonary shunt, PAH with small defect, or PAH after defect correction), anatomical-pathophysiological classification, date of diagnosis (i.e., date of first confirmatory cardiac catheterization), cardiac catheterization hemodynamic data (including mean right atrial pressure, mRAP; right ventricular end diastolic pressure, RVEDP; PAP; mPAP; PAWP; left ventricular end diastolic pressure, LVEDP; diastolic transpulmonary gradient, DPG (= PA diastolic pressure minus PAWP); Qp:Qs; PVR; and PVRi at baseline in room air and following acute vasodilator testing (AVT) were obtained from the retrospective chart review. Anatomical-pathophysiological classifications were based on the type of systemic to pulmonary shunt, as previously described. Simple pre-tricuspid shunts included atrial septal defect (ASD) and partial/total pulmonary venous return. Simple post-tricuspid shunts were ventricular septal defect (VSD), patent ductus arteriosus (PDA), and aortopulmonary window (APW). Combined shunts were combined pre- and post-tricuspid shunts or multiple systemic to pulmonary shunts. Atrioventricular septal defect (AVSD) was included in the combined lesions, for the purpose of this study. Lastly, complex CHD was defined if the patient had truncus arteriosus, transposition of great arteries (TGA), single ventricle with unobstructed pulmonary blood flow and pulmonary atresia, and VSD with major aorto-pulmonary collateral arteries.

### Follow-up and outcome measurement

The primary endpoint of the study was all-cause mortality. The secondary endpoint was combined mortality and WHO functional class or modified Ross heart failure worsening to class III–IV due to decompensated heart failure. Survival time was estimated from the date of cardiac catheterization diagnosis to the survival endpoint, which was taken either as the date of mortality or censoring. Patients were censored if they were lost to follow-up or alive on December 31, 2016.

### Statistics analysis

The patients’ baseline characteristics and outcomes were summarized using descriptive statistics. Normally distributed data was presented as the mean ± SD, or in cases where the distribution was not normal, as median with range. Categorical data was represented as number and percentage (%). Differences in the categorical data were assessed using Chi-square. The one-way analysis of variance with Bonferroni’s analysis was used to determine differences between data > 2 groups, for the continuous variables. Cumulative survival from date of diagnosis to the endpoint was calculated using the Kaplan-Meire method. The relation between baseline characteristics and mortality was evaluated with Cox regression and multivariate analysis. A *p*-value < 0.05 was considered to be statistically significant. The statistical analyses were performed with SPSS 19.0 for Windows (SPSS. Inc., Chicago, IL, USA).

## Results

### Patient characteristics

**Fig 2:**
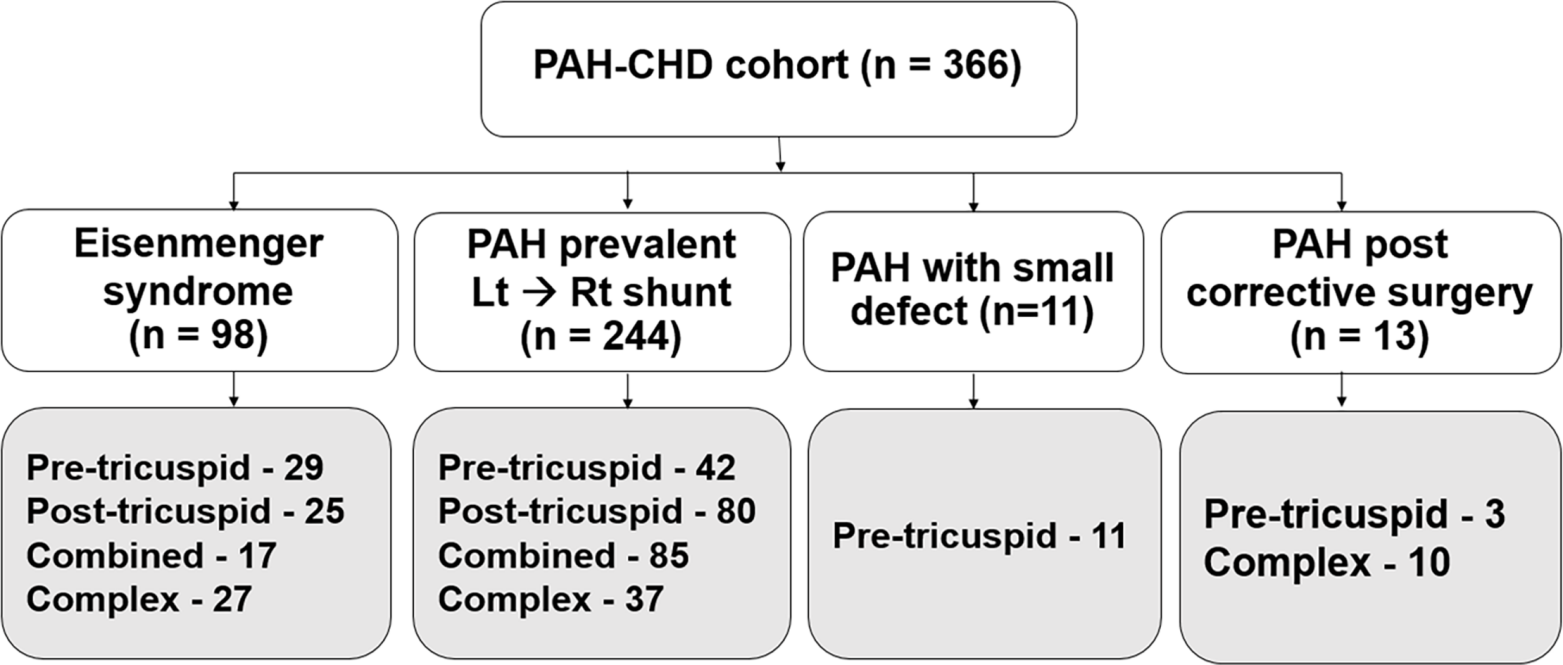
The study cohort based on clinical classifications. (PAH, pulmonary arterial hypertension; CHD, congenital heart disease).

Of the 460 patients in the PH-CHD database, 366 (age at cardiac catheterization diagnosis was 24.5 ± 17.6 years, 40% were male) were eligible to participate in this PAH-CHD study. Most of the patients had simple congenital systemic to pulmonary shunts and approximately 20% of the patient population had complex shunts (Fig 1). Clinical classifications identified patients as Eisenmenger syndrome (ES, 26.8%, n = 98), prevalent left to right shunt (66.7%, n = 244), PAH with small defect (3%, n = 11), or PAH after corrective surgery (3.5%, n = 13) (Fig 2).

The major clinical presentation was dyspnea and heart failure (62.5%, n = 229). Patients with trisomy 21 comprised 21% (n = 77) of the total PAH-CHD cohort. Hematocrit was 42.1 ± 6.4%. Considering the overall cardiac catheterization data for the 366 patients, mean RAP and RVEDP were 8.1 ± 6.25 and 10.5 ± 3.9 mmHg, respectively. The mPAP was as high as 64.8 ± 17.1 mmHg with DPG of 35.4 ± 16.0 mmHg. These values suggest that most of the patients in this series had combined pre- and post-capillary pulmonary hypertension. Qp:Qs was estimated to be 1.5 ± 1.0. PVR and the indexed PVR at the baseline hemodynamic was calculated to be 19.6 ± 14.2 WU and 15.9 ± 13.0 WU•m^2^, respectively. The ratio of PVR:SVR was 0.74 ± 1.1. Most patients (93%) also had AVT performed in the cardiac catheterization laboratory. The final mean PAP was measured to be 57.9 ± 8.4 mmHg, PVR was calculated to be 9.1 ± 9.8 WU (PVR index of 8.5 ± 9.9 WU•m^2^) and the PVR:SVR ratio was 0.5 ± 2.6 following AVT. The baseline characteristics for the four groups of clinical classifications and the four types of anatomical-pathophysiological classifications are shown in Tables 1 and 2. Both classifications identified differences in the baseline characteristics between the subgroups of PAH-CHD in the study. Patients with simple pre-tricuspid shunt were significantly older at diagnosis and females predominated, in comparison to simple post-tricuspid, combined, and complex shunts. Trisomy 21 was found in patients with post-tricuspid shunt and combined shunt more often than in patients with pre-tricuspid shunts or complex lesions. Considering the clinical classifications, patients with PAH with prevalent left to right shunts were younger than the patients with ES, PAH plus small defect, or PAH post-correction. The population of patients with ES in this cohort was comprised of mixed types of anatomical shunts, while patients with PAH with small defect were found to only have interatrial communication. PAH following corrective surgery was predominantly presented after complex congenital shunt repair. The data for hemodynamic cardiac catheterization showed the highest mPAP, DPG, and PVR in patients with ES, compared to the other groups from the clinical classifications.

**Table 1:** Patient demographics with anatomical-pathophysiological classifications (n = 366).

	Total (n = 366)	Pre-tricuspid shunt (n = 85)	Post-tricuspid shunts (n = 105)	Combined shunts (n = 102)	Complex shunts (n = 74)	*p*-value
Age at diagnosis (years)	24.5 ± 17.6	41.5 ± 15.7	24.1 ± 16.3	13.8 ± 12.1	19.9 ± 13.8	<0.001*
Male gender	145 (39.7%)	23 (27%)	32 (30.4%)	53 (51.9%)	37 (50%)	<0.001*
WHO functional class III-IV at diagnosis	49 (13.4%)	17 (20%)	10 (9.5%)	5 (4.9%)	17 (22.9%)	<0.001*
CHF at the presentation	229 (62.5%)	71 (83.5%)	33 (31.4%)	68 (66.6%)	57 (77%)	<0.001*
Trisomy 21	77 (21.0%)	2 (2.3%)	25 (23.8%)	50 (49%)	0	<0.001*
Hematocrit (%)	42.0 ± 6.7	42.6 ± 5.1	39.8 ± 6.5	40.4 ± 5.4	46.3 ± 7.4	<0.001*
Clinical classification						<0.001*
-ES	98 (26.8%)	29 (34.1%)	25 (23.8%)	17 (20%)	27 (36.5%)	
-Prevalent Lt♢ Rt shunt	244 (66.7%)	42 (49.5%)	80 (76.2%)	85 (80%)	37 (50%)	
-PAH-small defect	11 (3.0%)	11 (12.9%)	0	0	0	
-PAH post correction	13 (3.5%)	3 (3.5%)	0	0	10 (13.5%)	
Cardiac catheterization						
-mRAP (mmHg)	8.1 ± 6.2	8.8 ± 3.5	8.3 ± 4.2	7.6 ± 3.3	7.8 ± 3.7	0.64
-RVEDP (mmHg)	10.5 ± 3.9	11.4 ± 3.8	9.5 ± 4.4	10.2 ± 3.5	11.0 ± 3.8	0.004*
-mPAP (mmHg)	64.8 ± 17.1	58.4 ± 12.9	70.2 ± 16.6	62.3 ± 16.1	68.2 ± 20.0	<0.001*
-PAWP (mmHg)	10.8 ± 3.0	11.3 ± 2.7	10.4 ± 3.2	10.8 ± 3.2	10.8 ± 2.7	0.23
-DPG (mmHg)	35.4 ± 16.0	29.3 ± 11.5	40.3 ± 15.3	33.1 ± 16.3	38.6 ± 18.4	<0.001*
-Qp:Qs	1.5 ± 1.0	1.4 ± 0.7	1.7 ± 1.2	1.6 ± 1.0	1.3 ± 0.8	0.08
-PVR (WU)	19.6 ± 14.2	13.5 ± 10.2	19.6 ± 14.8	23.3 ± 14.1	21.2 ± 15.3	<0.001*
-PVR index (WU• m^2^)	15.9 ± 13.0	17.8 ± 12.9	17.6 ± 15.4	12.5 ± 9.7	15.8 ± 12.6	0.015

Data represented as mean ± SD, median (range) and n (% within column)

* Statistically significant at *p*-value < 0.05

WHO, World Health Organization; CHF, congestive heart failure; ES, Eisenmenger syndrome; Lt, left; Rt, right; PAH, pulmonary arterial hypertension; mRAP, mean right atrial pressure; RVEDP, right ventricular end diastolic pressure; mPAP, mean pulmonary arterial pressure; PAWP, pulmonary artery wedge pressure; DPG, diastolic transpulmonary gradient = difference of PA diastolic pressure and pulmonary arterial wedge pressure; Qp:Qs, flow to pulmonary and systemic ratio; PVR, pulmonary vascular resistance; WU, Wood Units.

**Table 2:** Patient demographics based on clinical classifications (n = 366).

	Total (n = 366)	ES (n = 98)	PAH-Lt to Rt shunt (n = 244)	PAH with small defect (n = 11)	PAH post correction (n = 13)	*p*-value
Age at diagnosis (years)	24.5 ± 17.6	30.7 ± 16.2	20.8 ± 17.1	35.5 ± 20.6	34.8 ± 15.3	<0.001*
Male gender	145 (39.7%)	31 (31.6%)	107 (43.8%)	1 (9%)	6 (46.1%)	0.03*
WHO functional class III-IV at diagnosis	49 (13.4%)	26 (26.5%)	17 (6.9%)	1 (9%)	5 (38.4%)	<0.001*
CHF at the presentation	229 (62.5%)	56 (57.1%)	155 (63.5%)	7 (63.6%)	11 (84.6%)	0.25
Trisomy 21	77 (21.0%)	14 (14.2%)	62 (25.4%)	1 (9%)	0	0.02*
Hematocrit (%)	42.0 ± 6.7	45.6 ± 7.1	40.3 ± 5.9	44.4 ± 3.9	45.2 ± 7.9	<0.001*
Type of shunts						<0.001*
-Pre-tricuspid shunts	85 (23.2%)	29 (29.5%)	42 (17.2%)	11 (100%)	3 (23.1%)	
-Post-tricuspid shunts	105 (28.7%)	25 (25.5%)	80 (32.7%)	0	0	
-Combined shunts	102 (27.9%)	17 (17.4%)	85 (34.8%)	0	0	
-Complex shunts	74 (20.2%)	27 (27.6%)	37 (15.1%)	0	10 (76.9%)	
Cardiac catheterization						
mRAP (mmHg)	8.1 ± 6.2	8.8 ± 6.8	7.6 ± 6.1	7.9 ± 2.5	11.1 ± 5.6	0.12
RVEDP (mmHg)	10.5 ± 3.9	11.3 ± 4.3	9.9 ± 3.6	11.3 ± 2.9	13.7 ± 5.1	<0.001*
mPAP (mmHg)	64.8 ± 17.1	75.8 ± 17.1	60.4 ± 14.7	61.6 ± 15.1	67.9 ± 19.7	<0.001*
PAWP (mmHg)	10.8 ± 3.0	11.1 ± 2.9	10.7 ± 3.0	10.4 ± 2.8	11.6 ± 3.2	0.51
DPG (mmHg)	35.4 ± 16.0	44.6 ± 15.9	31.9 ± 14.6	32.8 ± 13.1	34.5 ± 20.0	<0.001*
Qp:Qs	1.5 ± 1.0	1.0 ± 0.6	1.7 ± 1.0	1.1 ± 0.3	1.3 ± 0.6	<0.001*
PVR (WU)	19.6 ± 14.2	25.2 ± 17.1	17.7 ± 13.8	17.1 ± 9.3	13.7 ± 6.2	<0.001*
PVR index (WU• m^2^)	15.9 ± 13.0	27.1 ± 17.1	11.1 ± 7.2	20.2 ± 12.1	17.8 ± 8.4	<0.001*

Data represented by mean ± SD, median (range) and n (% within column)

* Statistical significance at *P*-value < 0.05

ES, Eisenmenger syndrome; PAH-Lt to Rt shunt, PAH with prevalent left to right shunt; WHO, World Health Organization; CHF, congestive heart failure; mRAP, mean right atrial pressure; RVEDP, right ventricular end diastolic pressure; mPAP, mean pulmonary arterial pressure; PAWP, pulmonary artery wedge pressure; DPG, diastolic transpulmonary gradient = difference of PA diastolic pressure and pulmonary arterial wedge pressure; Qp:Qs, flow to pulmonary and systemic ratio; PVR, pulmonary vascular resistance; WU, Wood Units.

### Survival of patients with PAH-CHD

**Fig 3:**
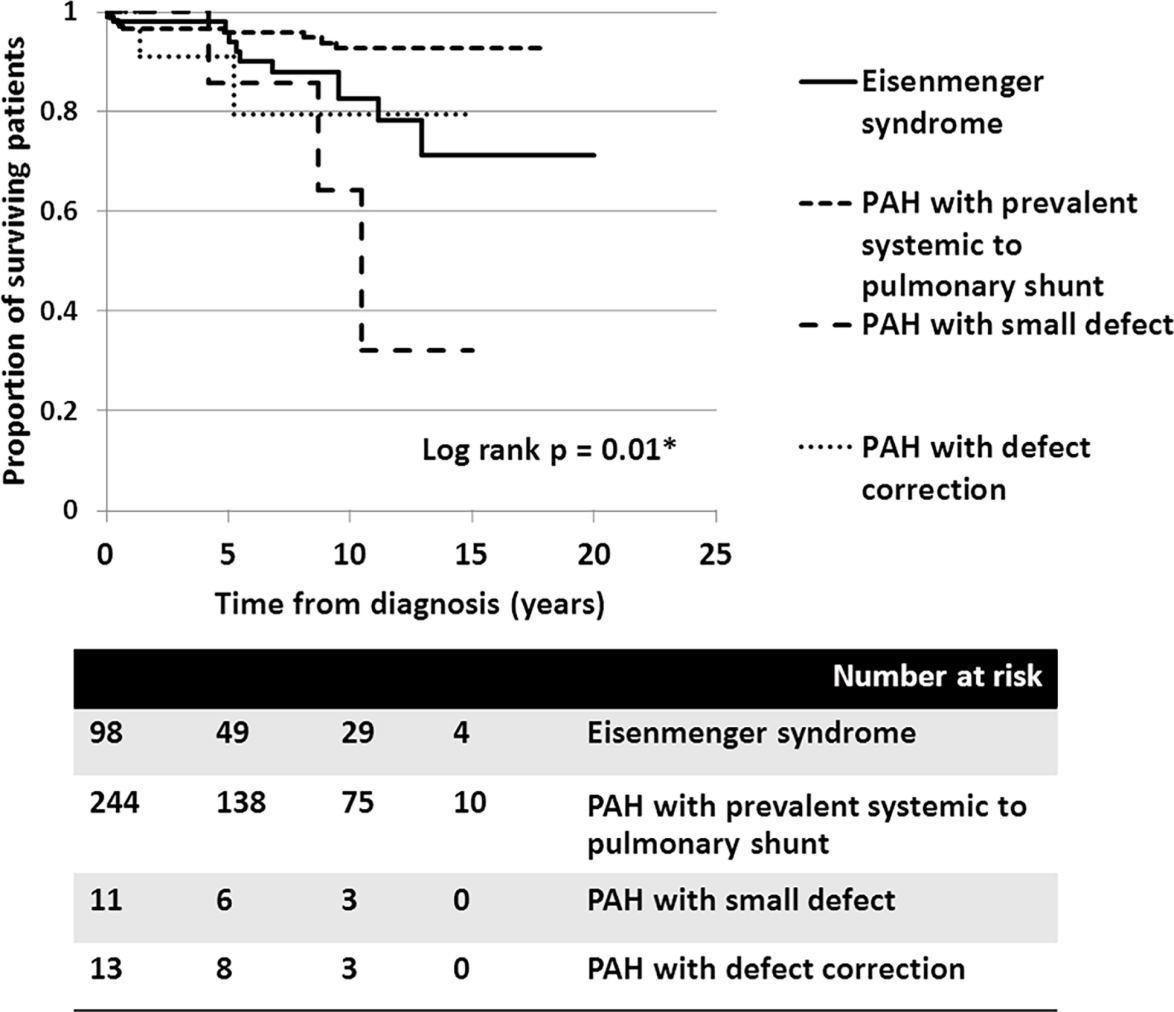
Cumulative survival of all PAH-CHD patients with different clinical classifications: 1) Eisenmenger syndrome (solid line), 2) PAH with prevalent systemic to pulmonary shunt (---), 3) PAH with small defect (^_ _ _^) and 4) PAH after defect correction (…).

At a median follow-up time of 5.9 years (0.1–20.7 years), 231 patients received interventions to their systemic to pulmonary shunt; 220 underwent total correction and 11 underwent fenestrated closure or one-way valve flap closure. Twenty-five patients who had PAH with prevalent left to right shunts decided not to be operated on for the lesion due to the primary physician’s and patient’s preferences. Of 366 patients, 203 patients received pulmonary vasodilators in follow-ups; 158 patients received monotherapy: phosphodiesterase-5 inhibitor (PDE5i, n = 71), prostaglandin analogue (PGI2 analogue, n = 84), endothelin receptor antagonist (ERA, n = 3). Forty five patients ultimately received combined therapy. Dual therapy was given to 35 patients (16 PDE5i + oral PGI2 analogue, 11 PDE5i + ERA, 6 PGI2 analogue + ERA, 1 PGI2 analogue inhalation + PDE5i, 1 PGI2 analogue inhalation + ERA). Triple therapy was given to 10 patients. Ninety-six patients received pulmonary vasodilators and underwent total defect correction. Of the 366 patients, 28 (7.6%) were deceased from the cohort (Fig 3).

All mortalities were primarily related to cardiac causes. Forty-eight patients (13.1%) were accounted for by combined mortality and declined functional class (to functional class III-IV) with decompensated heart failure at the most recent follow-up. In December, 2016, we were unable to contact 137 of the patients. The median time of follow-up for these patients was 2.1 years (0.1–14.1 years); therefore, the censoring was counted at the last date of follow-up in the survival analysis. Overall, the survival rate for patients with PAH-CHD at 5, 10, and 15 years was 95.3%, 88.6%, and 84.6%, respectively. Using clinical classifications, the survival analysis revealed that patients with PAH-small defect had inferior survivals, compared to those with ES, PAH after defect correction, or PAH with prevalent left to right shunt (*p* = 0.01) (Fig 3).

**Fig 4:**
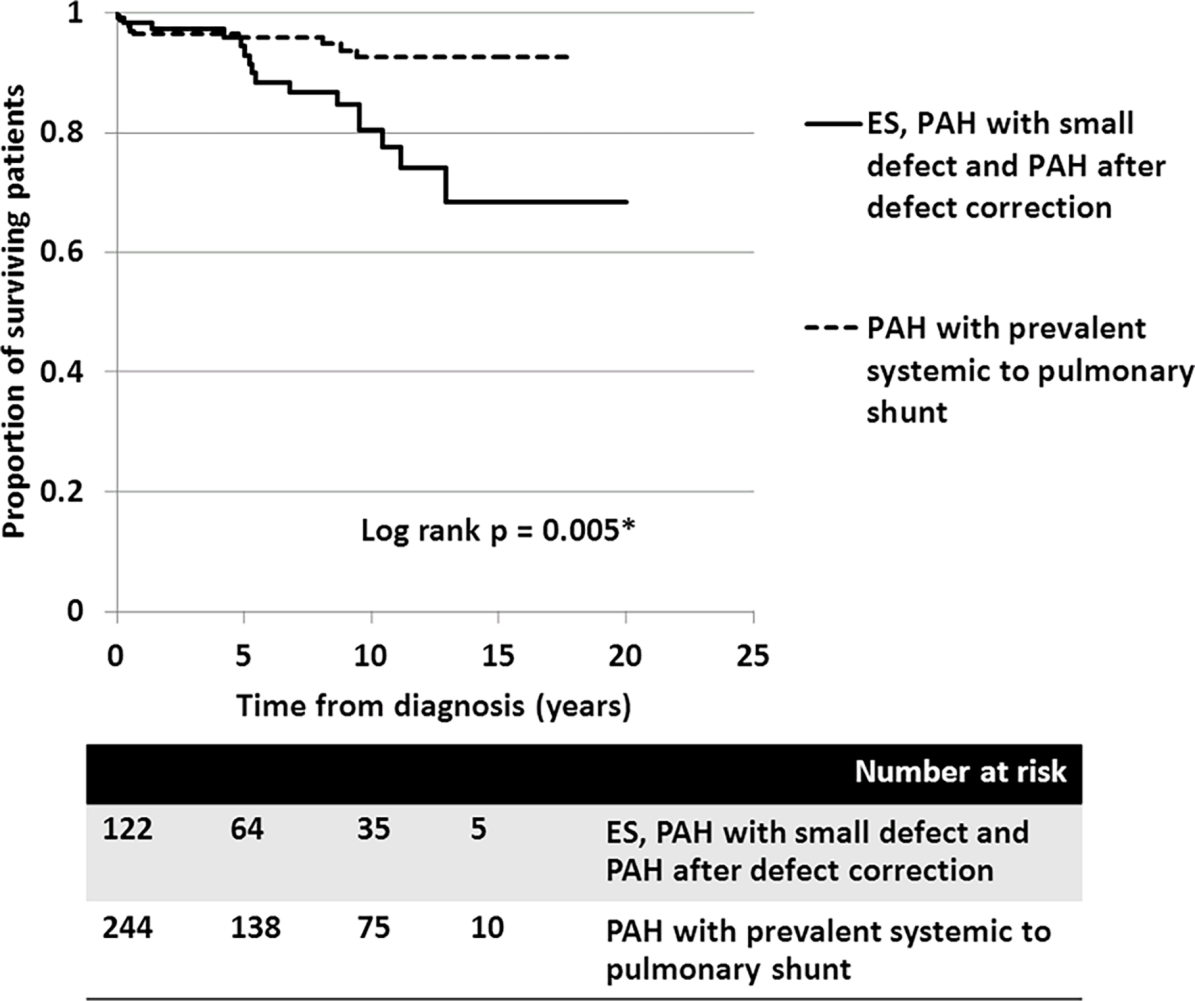
Cumulative survival of all PAH-CHD patients with different clinical classifications: 1) ES (Eisenmenger syndrome) + PAH-small defect + PAH after defect correction (solid line), and 2) PAH with prevalent systemic to pulmonary shunt (dashed line).

**Fig 5:**
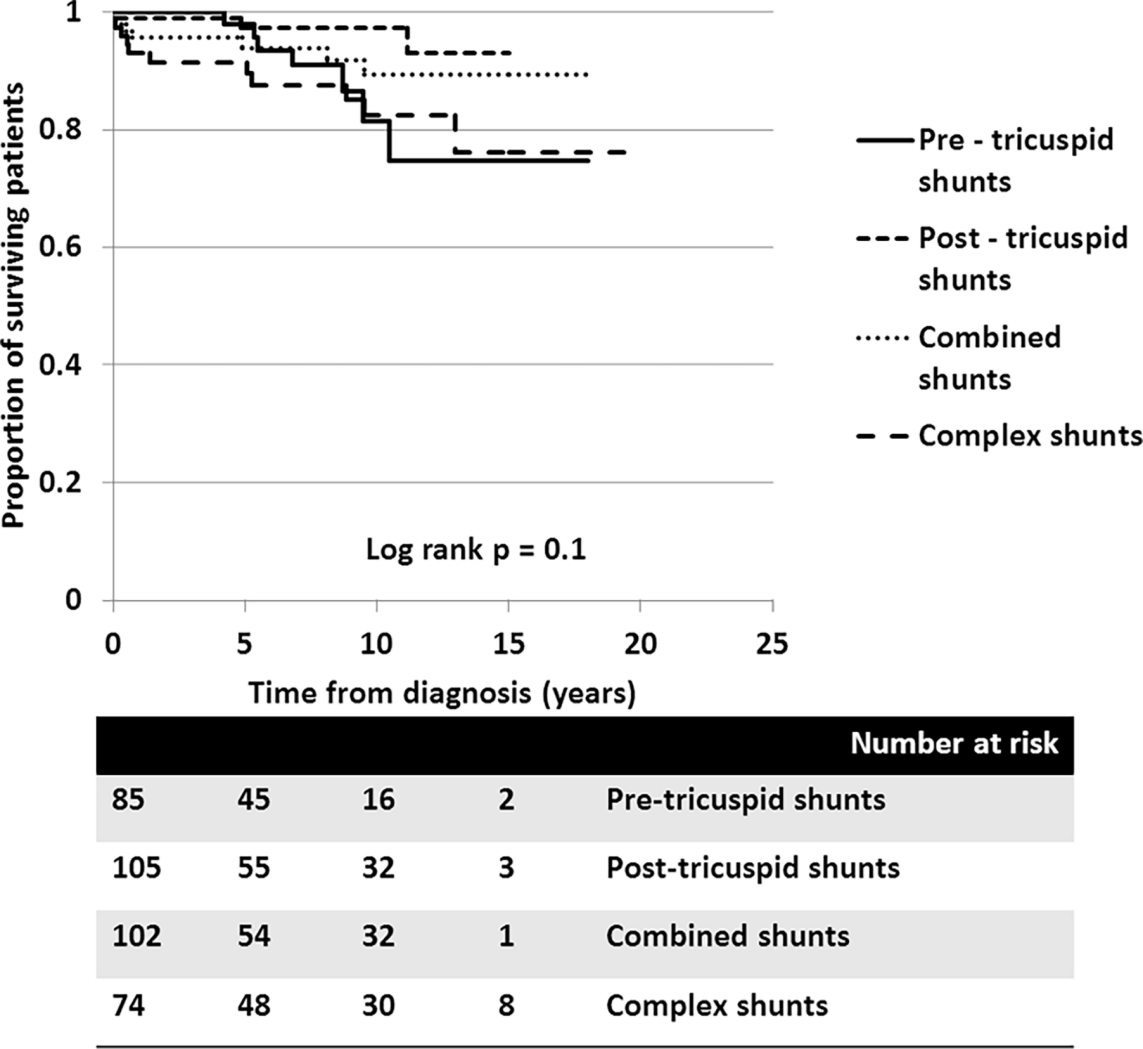
Cumulative survival of all PAH-CHD patients with different types of anatomical-pathophysiological classification: 1) Pre-tricuspid shunts (solid line), 2) Post-tricuspid shunts (---), 3) combined shunts (…), and 4) Complex shunts (^_ _ _^).

In the ES group, survival rates at 5, 10, and 15 years were 95.9%, 82.5%, and 71.1%, respectively. In patients with PAH-prevalent systemic to pulmonary shunts, the survival rates at 5, 10, and 15 years were 95.8%, 92.6%, and 92.6%, respectively. Despite patients receiving aggressive medical treatment, the worst survival rates, presented in PAH-small defect, at 5, 10, and 15 years were 85.7%, 64.3%, and 32.7%, respectively. Survival of patients with PAH following defect correction also showed fair survival rates at 5 and 10 years, of 90.9% and 79.5%, respectively. Consequently, the survival of patients who had PAH with prevalent systemic to pulmonary shunt was significantly better than those who did not (*p* = 0.005) (Fig 4). With regards to the anatomical-pathophysiological classifications, the survival curves showed no statistical difference between the different types (*p* = 0.1) (Fig 5). For the ES subgroup in this series, no significant difference in survival was seen between the four types of defect, which may have been due to the small sample population in each type (*p* = 0.6).

**Fig 6:**
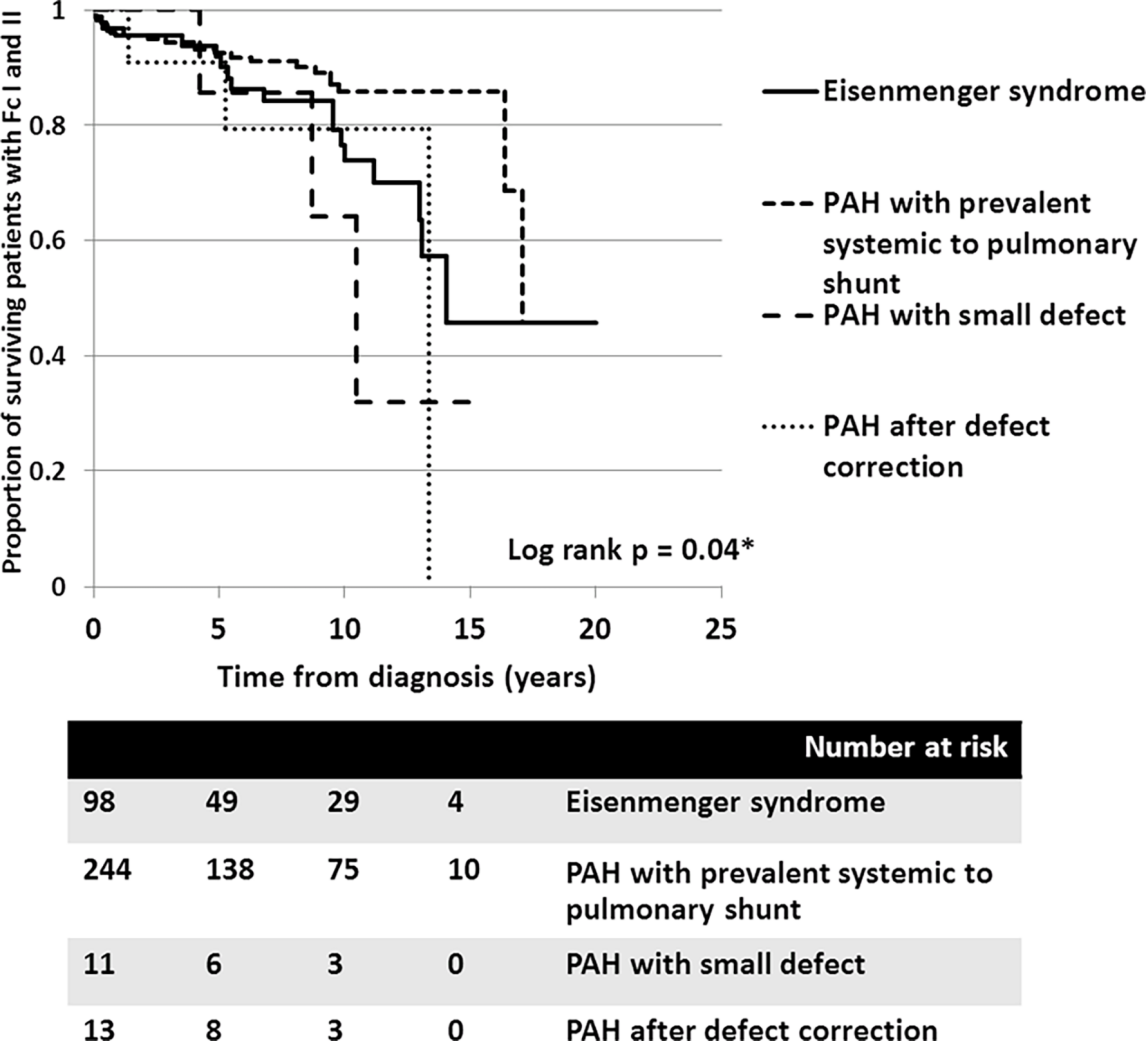
Cumulative survival rate free of functional class III to IV, due to decompensated heart failure in all PAH-CHD patients with different clinical classifications: 1) Eisenmenger syndrome (solid line), 2) PAH with prevalent systemic to pulmonary shunt (---), 3) PAH with small defect (^_ _ _^), and 4) PAH after defect correction (…).

**Fig 7:**
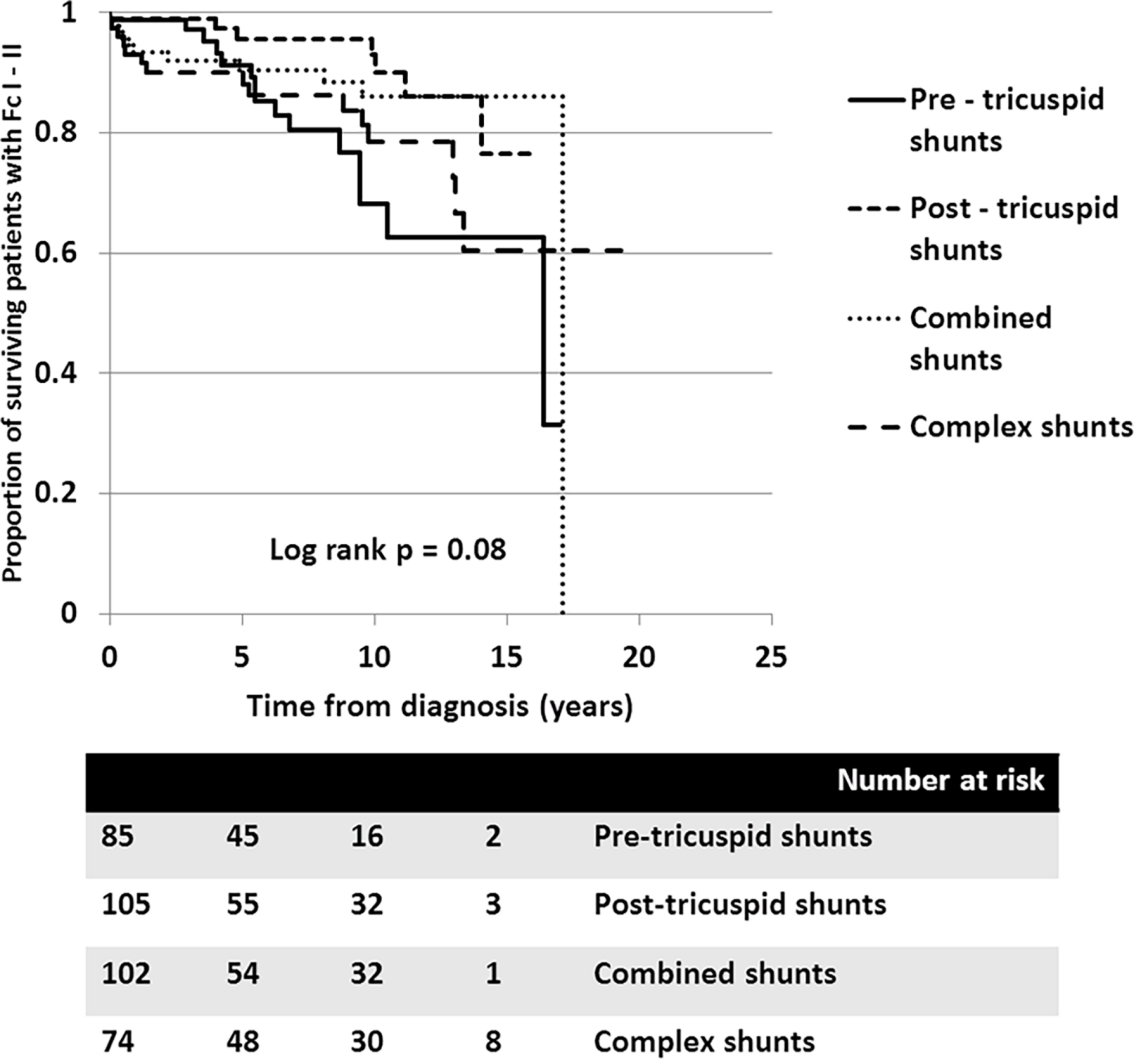
Cumulative survival rates free of functional class III to IV due to decompensated heart failure in all PAH-CHD patients with different types of anatomical-pathophysiological classification: 1) Pre-tricuspid shunts (solid line), 2) Post-tricuspid shunts (---), 3) Combined shunts (…), and 4) Complex shunts (^_ _ _^).

Looking at survival free of functional class declining to III-IV due to decompensated heart failure, the survival curves also show that the clinical classifications identified different survival rates for patients with PAH-CHD (*p* = 0.04). Fig 6 shows that the 10-year survival rate free of decompensated heart failure from diagnosis was more superior in patients with PAH-prevalent systemic to pulmonary shunts than for patients with ES, PAH after defect correction, or PAH with small defect. Again, no significance difference was seen in the survival rate free of decompensated heart failure among the four types of defects (Fig 7).

With regards to the different treatments, the survival rate of 220 patients who underwent defect closure at 5, 10, and 15 years was 95.4%, 91.5%, and 91.5%, respectively. Alternatively, the survival rate of 135 patients who were either not indicated for or refused to receive the intervention was 94.6%, 81.6%, and 68.4%, at 5, 10, and 15 years. The superior survival rate for the group undergoing operations (p = 0.02) suggests that the deliberate selection of patients for an intervention closure is crucial and may improve their survival outcomes. When comparing the survival of patients receiving a pulmonary vasodilator (n = 203) with those who did not (n = 163) no statistical difference was found between the groups (p = 0.13). This suggests that pulmonary vasodilators may improve the survival of patients who were indicated to receive the target therapy. In any case, the pulmonary vasodilators in this cohort were given to all patients with PAH after defect correction, most of the patients with ES, PAH with small defects and some patients with prevalent systemic to pulmonary shunts. The patients who did not receive a pulmonary vasodilator included a small numbers of patients with ES, PAH with a small defect and some patients with prevalent systemic to pulmonary shunt.

### Prognostic factors

The multivariate analysis showed five predictors of mortality in the PAH-CHD cohort, including WHO function class III-IV at the time of diagnosis (hazard ratio 2.5, 95% CI 1.1–6.1, *p* = 0.03), age at diagnosis < 10 years (hazard ratio 13.8, 95% CI 4.4–43.5, *p* < 0.001), PAH-small defect (hazard ratio 5.4, 95% CI 1.3–22.6, *p* = 0.02), elevated right atrial pressure > 15 mmHg (hazard ratio 10.6, 95% CI 1.9–59.1, *p* = 0.007), and baseline indexed PVR > 8 WU•m^2^ (hazard ratio 9.9, 95% CI 1.2–82.3, *p* = 0.03) (Table 3). In the Cox proportional hazard model that assesses predictors of the secondary endpoint: mortality and declined functional class to III-IV due to decompensated heart failure, four similar variables were found to be independently associated with the secondary endpoint in the multivariate analysis (Table 4). The variables included the WHO function class III-IV at the time of diagnosis (hazard ratio 2.6, 95% CI 1.2–5.4, *p* = 0.007), age at diagnosis < 10 years (hazard ratio 8.6, 95% CI 3.7–21.2, *p* < 0.001), elevated right atrial pressure > 15 mmHg (hazard ratio 8.8, 95% CI 2.2–34.3, *p* = 0.007), and baseline indexed PVR > 8 WU•m^2^ (hazard ratio 12.3, 95% CI 2.7–55.2, *p* = 0.001).

**Table 3:** Predictors of mortality.

Variables	Crude hazard ratio (95% CI)	*p-*value	Adjusted hazard ratio (95% CI)	*p-*value
Female gender	1.0 (0.5–21)	0.9		
Age at diagnosis **<** 10 years	4.0 (1.5–10.2)	0.003*	13.8 (4.4–43.5)	<0.001*
Presence of heart failure at initial diagnosis	4.1 (1.4–11.9)	0.008*	2.5 (0.8–7.4)	0.11
Functional class III-IV at time of diagnosis	4.0 (1.8–8.5)	<0.001*	2.5 (1.1–6.1)	0.03*
Presence of trisomy 21	0.2 (0.1–0.9)	0.04*	0.3 (0.1–1.8)	0.16
Presence of complex shunt	2.1 (0.9–4.4)	0.06	1.4 (0.6–3.2)	0.41
PAH-small defect	4.1 (1.2–13.3)	0.03**	5.4 (1.3–22.6)	0.02*
PAH after defect correction	0.5 (0.2–2.0)	0.33		
mRAP > 15 mmHg	8.2 (1.7–38.0)	0.007*	10.6 (1.9–59.1)	0.007*
PA diastolic pressure > 45 mmHg	1.7 (0.9–3.8)	0.15		
DPG > 30 mmHg	1.6 (3.7–4.9)	0.25		
Baseline PVRi > 8 WU•m^2^	10.2 (1.4–75.1)	0.02*	9.9 (1.2–82.3)	0.03*
Final PVRi > 8 WU•m^2^ post AVT	3.6 (1.6–7.8)	0.001*	2.1 (0.9–5.5)	0.1

Univariate analysis by chi-square test, Fisher’s exact test

Multivariate analysis by Cox regression

* Statistical significance at *p-*value < 0.05

PAH, pulmonary arterial hypertension; mRAP, mean right atrial pressure; PA, pulmonary artery; DPG, diastolic transpulmonary gradient = difference of PA diastolic pressure and pulmonary arterial wedge pressure; PVRi, pulmonary vascular resistance index; WU, Wood Units; AVT, acute pulmonary vasodilator testing.

**Table 4:** Predictors of combined mortality with declined functional class to III-IV due to decompensated heart failure.

Variables	Crude hazard ratio (95%CI)	*p-*value	Adjusted hazard ratio (95% CI)	*p-*value
Female gender	0.9 (0.5–1.6)	0.78		
Age at diagnosis < 10 years	3.3 (1.6–7.1)	0.002*	8.6 (3.7–21.2)	<0.001*
Presence of heart failure at initial diagnosis	2.3 (1.4–5.9)	0.004*	1.9 (0.9–4.1)	0.09
Functional class III-IV at time of diagnosis	3.7 (2.0–6.8)	<0.001*	2.6 (1.2–5.4)	0.007*
Presence of trisomy 21	0.5 (0.2–1.1)	0.11		
Presence of complex shunt	1.3 (0.7–2.5)	0.35	1.1 (0.6–2.3)	0.68
PAH-small defect	2.3 (0.7–7.5)	0.15	2.8 (0.7–10.5)	0.11
PAH after defect correction	0.5 (0.2–17)	0.25		
RAP > 15 mmHg	8.3 (2.4–29.0)	0.001*	8.8 (2.2–34.3)	0.002*
PA diastolic pressure > 45 mmHg	1.4 (0.8–2.5)	0.21		
DPG > 30 mmHg	1.2 (0.7–2.2)	0.5	0.3 (0.1–1.1)	0.07
Baseline PVRi > 8 WU•m^2^	10.1 (2.4–42.3)	0.001*	12.3 (2.7–55.2)	0.001*
Final PVRi post AVT > 8 WU•m^2^	2.8 (1.6–5.1)	<0.001	1.8 (0.9–3.7)	0.1

Univariate analysis by chi-square test, Fisher’s exact test

Multivariate analysis by Cox regression

* Statistical significance at *p-*value < 0.05

PAH, pulmonary arterial hypertension; mRAP, mean right atrial pressure; PA, pulmonary artery; DPG, diastolic transpulmonary gradient = difference of PA diastolic pressure and pulmonary arterial wedge pressure; PVRi, pulmonary vascular resistance index; WU, Wood Units; AVT, acute pulmonary vasodilator testing.

## Discussion

The present study used a 20-year congenital heart clinic database from a large, tertiary center in Thailand to report the survival of “real world” cases of PAH-CHD in the contemporary era. Of 366 patients (174 < 18 years of age; 192 ≥ 18 years of age), two-thirds still had clinical PAH with prevalent systemic to pulmonary shunts. Various types of lesions presented, including pre-tricuspid shunts (23%), post-tricuspid shunts (29%), combined shunts (28%), and complex shunts (20%). In this study, we found overall survival rates for patients with PAH-CHD at 5, 10, and 15 years to be 95.3%, 88.6%, and 84.6%, respectively, with a median time of follow-up of 5.9 years. Patients with ES had modest survival rates of 95.9%, 82.5%, and 71.1%, at 5, 10, and 15 years, respectively. These findings are comparable to recent studies [9, 10, 17]. When comparing the survival rates of different anatomical-pathophysiological classifications with clinical classifications, no statistical difference in survival was seen among the anatomical-pathophysiological shunts (*p* = 0.1). Conversely, clinical classifications at the initial diagnosis can identify differences in survival (*p* = 0.01). For the pair-wise comparisons, patients with PAH-prevalent left to right shunts showed a significantly superior survival rate compared to those with ES (*p* = 0.03) and PAH-small defect (*p* = 0.003). In this study, the estimated 10-year survival rates for patients with ES, PAH-prevalent systemic to pulmonary shunt, PAH with small defect, and PAH after defect correction were 82.5%, 92.6%, 64.3%, and 79.5%, respectively. To the best of our knowledge, this is one of a few studies to explore both the clinical classifications and the anatomical-pathophysiological classifications with regards to survival of all PAH-CHD patients. Moreover, the clinical classifications seem able to identify the survival prognosis of the patients.

True cases of PAH-CHD are often difficult to identify because of the unknown exact time of PAH and time of presentation. Moreover, the congenital systemic to pulmonary shunt is a congenital malformation affecting the pulmonary vasculature from childhood, and the effects may continue into adulthood if the lesion is left untreated. Since some patients with this disease die during childhood, this study includes “prevalent cases,” with the inclusion of both children and adults, based on the 2013 PAH definition [2, 3]. In this study, patients with PAH with prevalent left to right shunts appear to be younger than those with ES, PAH with small defect, or PAH post-correction. The vast majority of PAH-prevalent left to right shunts were combined lesions and post-tricuspid lesions. In addition, patients with combined systemic to pulmonary shunts tended to be the youngest at diagnosis, followed by patients with complex shunts or post-tricuspid shunts, suggesting that different types of cardiac anatomy affect the natural history and presentation [13, 21]. Pre-tricuspid lesions commonly present late in adulthood leading to a long-standing elevation in pulmonary pressure [6, 8, 21]. Nonetheless, in this study, half of the patients with pre-tricuspid lesions continued to have prevalent left to right shunts, while 34% of the patients were classified as having ES. Complex shunts can be diagnosed early as they are usually visibly cyanotic. Nevertheless, in this study, one-third of the patients with complex shunts were identified as ES at the time of diagnosis. The treatment of patients was either medication plus defect correction or medical therapy only, given the clinical classification and based on the multidisciplinary team conference. In fact, 75% of the patients in this cohort who were targeted for PAH medical therapy received monotherapy, because of the lack of resources for healthcare.

The survival rates of patients with PAH have been published on the basis of several large national registries. REVEAL, French, Chinese, UK, and Ireland registries have illustrated the significant improvements in survival of patients with idiopathic PAH, in comparison to the original NIH registry [10, 15–17, 19, 22]. For patients with PAH-CHD, the survival rate has been reported to be better than that of idiopathic or hereditary PAH [9, 17, 23, 24]. Nevertheless, some patients of specific PAH-CHD subclasses show survival rates that are similar to those of idiopathic PAH [18, 20]. Recent studies have found improved five-year survival rates for patients with ES up to 91–95%, which is consistent with our study [9, 11, 12, 25]. This may be due to the consequences of PAH-targeted therapy in the contemporary period. Marnes and colleagues [9] classified 192 patients with PAH-CHD in their center with recent clinical classifications and showed that the estimated survival rates for patients with ES, PAH-prevalent systemic to pulmonary shunts, and PAH after defect correction were 87%, 86%, and 36%, respectively, at 20 years. The survival rate of patients with PAH-small defect was 66% at 15 years. Likewise, for 240 PAH-CHD patients with ES, PAH with small defect, or PAH after defect correction from the REHAP registry [10], ES had a better survival rate than PAH after defect correction, but no statistically significant difference was found with regards to small defects. We found a similar trend in that the best survival seems to be present in patients with PAH-prevalent systemic to pulmonary shunts, while the worst survival was found in patients with PAH with a small defect, followed by PAH after defect correction. Because of the small populations of patients with PAH-small defect and PAH after defect correction, no statistically significant differences were seen between ES, PAH-small defect, and PAH after defect correction. In addition, all patients with PAH-small defect in this study had pre-tricuspid lesions, which may be a questionably small defect in idiopathic PAH patients. The survival rates for patients with PAH-small defect are therefore comparable to the survival rates of patients with idiopathic PAH, reported previously [10,17]. These results are based on a small sample size of patients who had PAH with small defect and should be interpreted cautiously.

In ES, the evidence suggests that the defect location has prognostic implications according to differences in the evolution of pulmonary vascular occlusive disease among pre-tricuspid, post-tricuspid, and complex lesions [11, 12]. Recently, a large cohort of patients with ES [12] demonstrated a lower 5-year survival in the pre-tricuspid group (55.6%) compared to the post-tricuspid (76.6%, *p*<0.001) and complex-shunt group (71.4%, *p* = 0.011). This implies that anatomical-pathophysiological classification can identify survival in ES patients, similarly to the pivotal study by Diller GP et al. [11]. Nevertheless, no significant difference in 5-year survival was found between the post-tricuspid and the complex-lesion groups in patients with ES. We also used anatomical-pathophysiological classifications to categorize all PAH-CHD patients in our study, but no differences were seen in survival, either survival free of functional class worsening or mortality (*p* = 0.1). This was based on two-thirds of the patient population in the present study who had PAH with prevalent systemic to pulmonary shunt. This finding is in contrast to the finding of Ramjug and colleagues [13], who were first to apply both classifications to their PAH-CHD patients and concluded that only the anatomical-pathophysiological system identified the patients’ survival from the point of referral.

Various prognostic factors for patients with PAH-CHD have been described in previous publications. Diller and colleagues [11] reported that patients with ES with a complex anatomy had a significantly worse prognosis when compared to patients with a simple anatomy, and a worse functional class. The signs of heart failure were found to be predictive of death in patients with ES. The REVEAL registry illustrated four variables associated with improved survival of patients with PAH-CHD at four years from enrollment: longer 6-minute walk distance, lower mean right atrial pressure, brain natriuretic peptide level < 50 pg/mL, and the presence of acute vasoreactivity [18]. Patients in the WHO functional class III-IV at presentation have been identified as having a mortality risk similar to that of the overall PAH-CHD in the REHAP registry [10]. Baseline N-terminal pro-brain natriuretic peptide > 500 ng/L and tricuspid annular plane systolic excursion < 15 mm were also found to be predictors of mortality in 91 adults with PAH-CHD by Schuuring et al. [26]. Older age, higher creatinine levels, lower transfer factors in the lung for carbon monoxide percentage predicted, and non-post-tricuspid shunts were found to be independent risks for poor survival outcomes by Ramjug et al. [13]. Recently, serial change in the WHO functional class, arterial saturation at peak exercise, six-minute walk distance, N-terminal pro-brain natriuretic peptide, and tricuspid annular plane systolic excursion were described as being predictive of mortality in 92 patients with PAH-CHD [27]. In our study, independent risks of mortality include functional class worsening, WHO function class III-IV at the time of diagnosis, age at diagnosis < 10 years, elevated right atrial pressure > 15 mmHg, and baseline indexed PVR > 8 WU•m^2^. Gender, trisomy 21, presence of complex congenital shunts, PAH after defect correction, diastolic transpulmonary gradient, and diastolic PA pressure were not shown to be significant risk factors in the multivariate analysis. A worsening WHO functional class and elevated right atrial pressure at the time of diagnosis are often associated with right ventricular failure, which increases mortality, according to several reports [10, 11, 18]. In our study cohort, age at the time of diagnosis < 10 years was identified as a risk, which may be explained by the population of children with PAH-CHD being included in this study, and by the fact that some of the young children became deceased after their diagnosis and treatment. The survival of children with complex CHD and ES may be worse than that reported for adults, which is also consistent with earlier reports [24]. This finding emphasizes the need for physicians to be aware of patient operability and the risk of poor outcomes in young children.

## Study limitations

In this retrospective-cohort and observational study with prevalent cases, selection bias is unavoidable. We attempted to strictly select patients with PAH-CHD who had been newly diagnosed and had undergone cardiac catheterization to confirm that their diagnoses of PAH were according to the current PAH definition [2–4]. We excluded patients who had staged repaired single ventricle due to the unique characteristics of this group. Mixed populations of children and adults were included, though the children were at least three months old, in keeping with the current definition of pediatric PAH. With regards to the mixed age group (174 age < 18 years; 192 age ≥ 18 years), an additional survival analysis of children (age < 18 years) and adults was conducted. No statistic difference was seen between the age groups (p = 0.89) (S1 Fig). Our retrospective data lacked some variables that could have been prognostic factors, such as brain natriuretic peptide level, six-minute walk distance, and tricuspid annular plane systolic excursion. In any case, complete cardiac catheterization data was provided for the baseline characteristics. To deal with the immortal time bias, all patients that had fulfilled the criteria were included, and for the survival analysis, the time of cardiac catheterization diagnosis was counted as time 0. The survival endpoint was taken as either the date of mortality or censoring. All patients were contacted in 2016 to confirm their functional class and their status as being deceased or alive. We were unable to contact 137 patients in December 2016, and for the survival analysis, censoring was counted as the last date of follow-up. The median time for follow-up in these patients was 2.1 years (0.1–14.1 years). This database for PH-CHD was from a single center, intended to serve as the preliminary data for a national registry. The data from a large aggregated cohort still needs to be collected over a longer period to gain more fruitful knowledge for managing PAH-CHD.

## Conclusions

We report a modest long-term survival of patients with PAH-CHD, based on a dataset from a large, single-center cohort in Thailand. At the median time of 5.9 years, the overall estimated survival rates for patients with PAH-CHD at 5, 10, and 15 years were 95.3%, 88.6%, and 84.6%, respectively, and the best survival rate was found for patients with PAH with prevalent systemic to pulmonary shunts. Different anatomical-pathophysiological shunts affect the natural presentation, while clinical classifications indicate treatment strategies and long-term outcomes. The independent predictors of mortality and functional class worsening were found to be the WHO function class III-IV at the time of diagnosis, age at diagnosis < 10 years, elevated right atrial pressure > 15 mmHg, and baseline indexed PVR > 8 WU•m^2^. Advanced therapies in the contemporary era can improve the survival rates of deliberately selected patients.

## Supporting information

S1 Fig Cumulative survival of all PAH-CHD patients in different age groups: Children age < 18 years (solid line) and adults age ≥ 18 years (dashed line). (DOCX)
