# Supplementary figures and images for: Contemporary survival of patients with pulmonary arterial hypertension and congenital systemic to pulmonary shunts
Source: PLoS One. 2018 Apr 17;13(4):e0195092. doi: 10.1371/journal.pone.0195092 (PMC5903600; doi:10.1371/journal.pone.0195092)

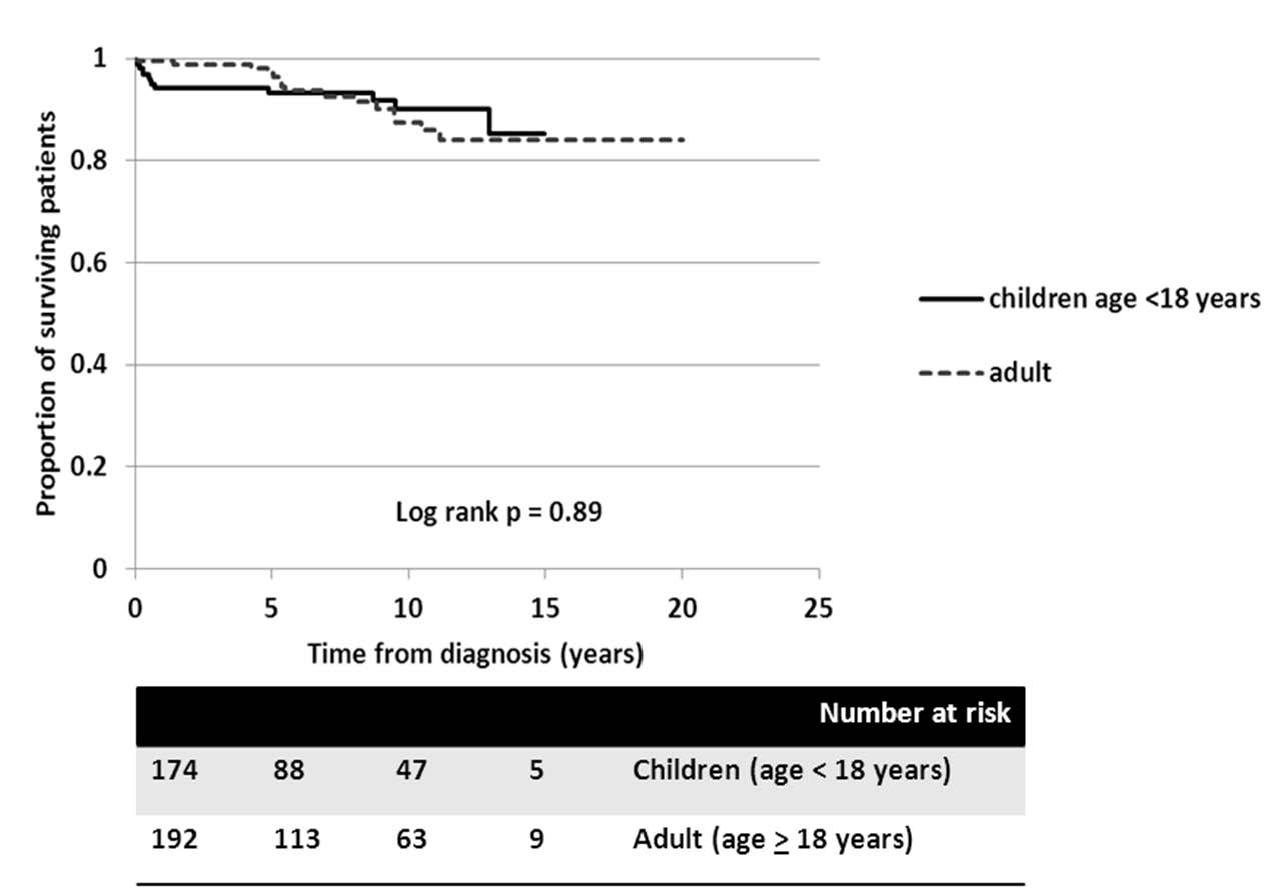

Supplement: S1 Fig — (DOCX) [file pone.0195092.s001.docx]
